# Vitamin Bullets. Microencapsulated Feeds to Fortify Shellfish and Tackle Human Nutrient Deficiencies

**DOI:** 10.3389/fnut.2020.00102

**Published:** 2020-07-20

**Authors:** David F. Willer, David C. Aldridge

**Affiliations:** Aquatic Ecology Group, Department of Zoology, University of Cambridge Conservation Research Institute, University of Cambridge, Cambridge, United Kingdom

**Keywords:** nutritional fortification, microcapsules, aquaculture, bivalve shellfish, nutrient deficiencies, vitamins, human health, oysters

## Abstract

Over two billion people worldwide are micronutrient deficient, with regionally specific deficiencies. Fortification of food with micronutrients has become an industry standard for enhancing public health. Bivalve shellfish (e.g., oysters, clams, and mussels) provide the most sustainable source of animal protein on the planet, and the market is rapidly growing—with production in China increasing 1,000-fold since 1980 to an annual 36 kg capita^−1^ consumption level. Bivalves are also unique in that micronutrients consumed at their end-life stage will be digested by humans, as humans consume the entire organism including the gut. We have developed a novel microencapsulated vehicle for delivering micronutrients to bivalves, tailored for optimal size, shape, buoyancy, and palatability, demonstrating the potential of fortified bivalves to tackle human nutrient deficiencies. Oysters fed vitamin A and D microcapsules at a 3% initial dosage for just 8 h had elevated tissue vitamin content. A serving of just two such bivalves provides enough vitamin A and D to meet human dietary RDAs. Scale-up of this technology and application to other bivalve species including clams and mussels could provide a low-cost and highly sustainable mechanism to contribute toward tackling nutrient deficiencies globally.

## Introduction

The World Health Organization (WHO) estimates over two billion people worldwide are micronutrient deficient (1). Vitamin A and D deficiencies are of particular concern (2), with 33% of children and one in six pregnant women lacking sufficient vitamin A (1, 3). Regional deficiencies can be especially pronounced. In Ghana more than 76% of children are vitamin A deficient, causing widespread mortality and blindness (1, 2). In India 85% of citizens are vitamin D deficient, causing cardiovascular diseases, osteoporosis, and rickets (4–6). Even in the US over 40% of the population is vitamin D deficient (7). Here we demonstrate a cheap and effective way of integrating micronutrients into the food supply, thus representing a highly efficient and attractive way to help tackle a major human health challenge (5).

Delivering micronutrients to the human population through animal products offers major advantages. Nutrients important to human health are less bioavailable in plants than meat, and rising atmospheric CO_2_ content is reducing the absolute concentration of these nutrients in plants (8, 9). Nutrients consumed alongside the muscle and fat of an animal are also more bioavailable to the human digestive system than nutrients in a supplemental pill (10). Fat must be present in the digestive tract for essential fat-soluble vitamins such as A, D, E, K, and carotenoids to be absorbed, and muscle protein breakdown enhances absorption of key micronutrients including iron concurrently present in the gut (11–13). In addition, alternatives such as vitamin supplements or fortified food condiments are often expensive and seen as a luxury by the people who really need them (5). Given that the global regions where vitamin deficiencies are most prevalent also tend to be the poorest, targeted integration of nutrients directly into the food supply (e.g., in rice and milk) has become important and commonplace. Costs are comparable or lower than providing a supplemental pill, and compliance is easier; poor consumers will continue to buy their now marginally more expensive food whereas they are unlikely to make an additional purchase to buy supplements (2, 5, 14). However, current animal meat production methods are causing catastrophic environmental damage, driving 15% of greenhouse gas emissions and widespread biodiversity loss (15). There is an urgent need for a sustainable alternative.

Bivalve shellfish, such as clams, oysters, mussels, and scallops, are a highly attractive yet underutilized food source with the capacity to provide the global population with key nutrients. Bivalves have a higher protein content than beef, are a rich source of omega-3 fatty acids, and have some of the highest levels of key minerals of all animal foods (16). They are also very sustainable to farm, having a far lower environmental footprint than animal meat or fish, and lower even than many plant crops such as wheat, soya, and rice (17). Bivalves are a highly affordable food source in nations where they are produced at large scale, such as China (18). There is great potential to sustainably expand bivalve aquaculture worldwide, with over 1,500,000 km^2^ available for sustainable low-cost industry development, particularly around the west coast of Africa and India (19). In areas including the Malabar and Goa coasts of India bivalves such as the green mussel (*Perna viridis*) are already staple foods for poor populations (18, 20). However, whilst bivalves are nutrient rich the level of nutrients they deliver naturally is unlikely to solve global nutrient deficiencies. Innovations in bivalve production can change this.

The “depuration” stage of bivalve production, during which bivalves are held in cleansing tanks for 48 h after harvest, represents a unique opportunity for integrating nutrients into the bivalve gut and surrounding tissue. As humans consume the entire organism including the gut when they eat a bivalve, these nutrients will be available to humans (21). In other animals, supplemental nutrients can be included into the feed, but this method is inefficient because feeds must be fed to animals for a far longer period of the animals' lifetime in order to generate elevated nutrient levels in the animals' tissue (22, 23). Micronutrient fortification during the depuration stage could allow the levels of a specific nutrient such as vitamin A or D to be increased in the food supply to meet specific regional needs. As bivalves also tend to be consumed locally (18), this would be a highly efficient and targeted method to tackle nutrient deficiencies. There is however a need for a method to deliver micronutrients to bivalves during depuration.

Novel microencapsulated feeds developed through recent chemical engineering innovations can provide a delivery vehicle for micronutrients to bivalves (24). It has already been demonstrated that this form of microcapsules are digestible by bivalves and can improve bivalve growth and sexual maturation [(25, 26); Willer and Aldridge, in review]. Mass production is simple and cost-effective (24, 26), and the dry microcapsules have shelf lives in excess of one year in any sealed dry container (e.g., mylar bags) thus circumventing conventional feed wastage costs (27). Capsule characteristics are designed to maximize feeding efficiency (27) and minimize nutrient leaching to water (28–30). The specific nutritional content of the microcapsules can easily be tailored. For depuration, this makes it possible to create microcapsules containing only the micronutrients required by the human population for fortification, without any other food, minimizing the overall quantity of microcapsules required.

This investigation aimed to formulate and characterize a new form of micronutrient microcapsules, find out whether bivalves would consume them, and whether this would lead to elevated micronutrient levels in bivalve tissue. We also aimed to determine the optimum concentration and timeframe for delivering microencapsulated micronutrients to bivalves, and how the resultant micronutrient levels in bivalve tissue would compare to human Recommended Daily Allowances (RDAs) and other foods. Microcapsules fortified with vitamins A or D were selected as a case study, due to the prevalence of vitamin A and D deficiencies worldwide. Pacific oysters (*Crassostrea gigas*) were used as a case bivalve species, due to their widespread popularity as a food source, worth $USD 6.7 billion in 2017 (18). The natural diet of these oysters is phytoplankton between 10 and 400 μm (31). Our target size microcapsule to develop was around ~100 μm—small enough to avoid excessive rejection in psuedofeces but with enough mass to allow relatively long retention times in the stomach (31). The microcapsules also needed to have a rough surface texture to facilitate uptake and a neutral or slightly negative buoyancy to maximize uptake into the inhalant current (24, 27).

## Materials and Methods

### Microcapsule Manufacture

Lipid-walled microcapsules containing vitamin A at retinyl acetate at 200 mg g^−1^ or vitamin D as cholecalciferol at 20 mg g^−1^ were manufactured under patent by BioBullets (BioBullets Ltd., Cambridge, UK) (11, 32). The remainder of the weight consisted the vegetable oil-based encapsulant and lipid-based bulking agents. To manufacture the particles a premix slurry containing the waxy encapsulant, bulking agents, and the powdered vitamin were prepared under conditions of controlled shear. The slurry was pumped into an ultrasonic atomizing nozzle at the top of a cooling chamber. The atomized particles formed near-perfect spheres as they cooled and fell to the chamber base. Further particle cooling was achieved with an air-conveying system before discharge via cyclone to a fluid bed processor. The encapsulated particles were then coated with a proprietary non-ionic surfactant to aid dispersion in water. Further cooling in the fluid bed removed all heat of crystallization from the microparticles before packaging. All components of the formulation were food grade.

### Microcapsule Characterization

Scanning electron microscopy (SEM) was used to examine the morphology of complete vitamin A and D microcapsules, and microcapsules freeze-fractured using liquid nitrogen and a cold hammer. The entirety of a 1 g sample was mapped for each vitamin, and then a representative selection of SEM images were taken using an FEI Quanta 650F (Thermo Fisher Scientific, USA) under high-vaccum and 3 kV. A Malvern Mastersizer 3000 (Malvern Panalytical, UK) was used to assess the particle size distribution of microcapsules. Five samples of both vitamin A and D microcapsules were analyzed. The Mastersizer 3000 generated fitted size distribution curves for each microcapsule type, alongside mean particle size, and residual standard deviation.

### Bivalve Nutritional Fortification

Bivalve nutritional fortification experiments were undertaken at the University of Cambridge UK in December 2019, under conditions to simulate commercial depuration protocols. Experiments were carried out in a controlled temperature room held at 15°C, in constantly aerated tanks each containing 1 L of artificial seawater at salinity 30‰ (H2Ocean Aquarium Salt, D-D The Aquarium Solution Ltd., UK) (21, 33). Each tank contained one adult *Crassostrea gigas* oyster, size grade AA, received directly from commercial depuration tanks at Colchester Oyster Fishery, UK. The mean dry weight (dw) of these grade AA oysters was obtained from 20 samples at 1.88 ± 0.11 g. Each oyster was fed a 50:50 blend of both vitamin A and D microcapsules at doses and timeframes feasible during the 48-h depuration period. There were 105 individual tanks, allowing for five biological replicate oysters to be fed microcapsules at doses of 3, 6, and 9% (33) dw feed per dw oyster over 2, 4, 8, 16, and 32 h (21), alongside 0 and 32 h controls at doses of 0%. Feed concentrations refer to the initial quantity of feed given at time = 0, no feed was added to the tanks during the remainder of the course of the experiments. At the end of each timeframe, each oyster was immediately removed from its tank. Oysters were then shucked and any water inside the shells was drained off. The entire soft tissue of each oyster was then removed and frozen at −80°C (34).

### Bivalve Vitamin A and D Analysis

The total vitamin A and D content of entire oyster soft tissue samples was measured by a UKAS accredited analytical service [Premier Analytical Services (PAS), UK]. PAS are also regulated by external quality performance testing (FAPAS and LGC schemes) to demonstrate the accuracy of their results. Samples were delivered to PAS from Cambridge within 4 h under dry ice. All five biological replicates for each dose and timeframe sample type were pooled into a single compound sample during the analysis. Each sample run included a control sample with established control limits that had to be met for the run to be passed, alongside spiked samples for which the recovery of these also had to be within acceptable limits.

Vitamin A was determined as the sum contribution of retinol and carotenes, and the limit of detection (LOD) was 10 μg 100 g^−1^. Measurement of retinol followed UKAS protocol C-TM-021; retinol was saponified with alcoholic KOH and extracted into hexane, then the cis and trans isomers the determined using High-performance Liquid Chromatography (HPLC) with UV detection at 325 nm (35). Measurement of carotenes followed UKAS protocol C-TM-087; samples were saponified with alcoholic KOH and carotenes extracted into hexane, then the alpha- and ß-carotenes were determined using reverse-phase HPLC with visible detection (36). Vitamin D was determined as the sum of vitamins D2 and D3 following UKAS protocol C-TM-273, and the limit of detection was 0.3 μg 100 g^−1^. Vitamin D2 (ergocalciferol) and vitamin D3 (cholecalciferol) were saponified with alcoholic potassium hydroxide and extracted into hexane/diethyl ether, then the vitamin D2 and D3 were measured using HPLC with UV detection (37).

The output data consisted of a single compound measurement of vitamin A or D for each dose and timeframe sample type. Relative uncertainty in the measurements was calculated as 2× standard deviation/mean value from quality control tests run immediately before our sample set. The relative uncertainty (RU) for the vitamin A data points was 12.6% and for the vitamin D data points 19.6%. A statistical analysis was not appropriate as biological replicates were pooled for analysis to give the single compound measurement for each sample type. Pooling was necessary due to limits of detection and practical constraints, and followed a widely used approach for such analyses (38–40). Dose response curves were then plotted for both the vitamin A and D microcapsules (**Figure 3**). The yield, or percentage of microcapsules in the oyster sample in relation to the total amount in the tank, was also calculated for each oyster sample (Supplementary Information; **Figure 3**, Raw Data).

## Results

### Characteristics of Micronutrient Microcapsules

**Figure 1:**
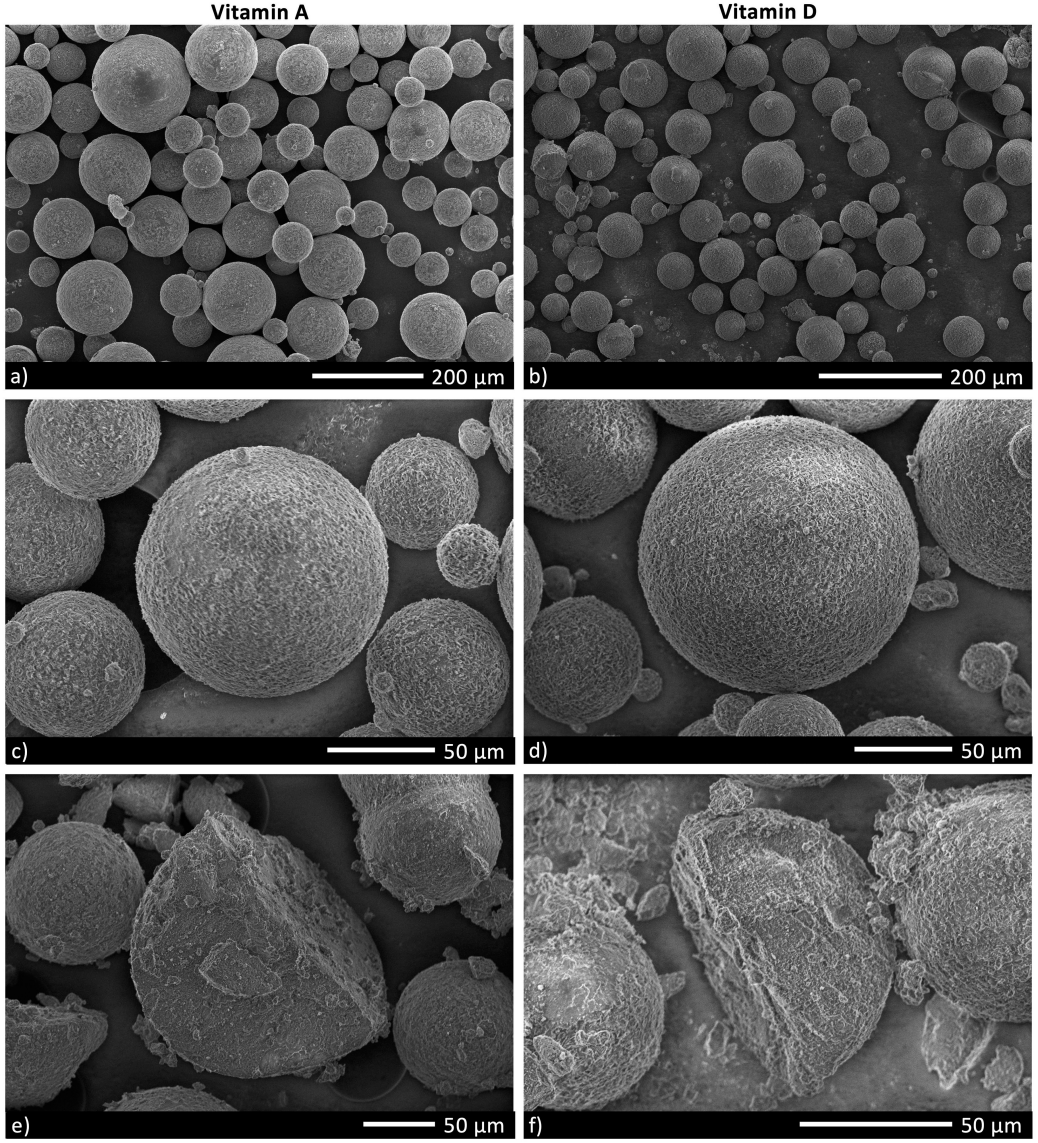
Scanning electron microscopy (SEM) images of Vitamin A and D microcapsules. **(a,b)** Demonstrate the typical variation in morphology in a sample of microcapsules. **(c,d)** Are close-up images of individual microcapsules. The microcapsules in **(e,f)** have been freeze-fractured to visualize internal structure.

**Figure 2:**
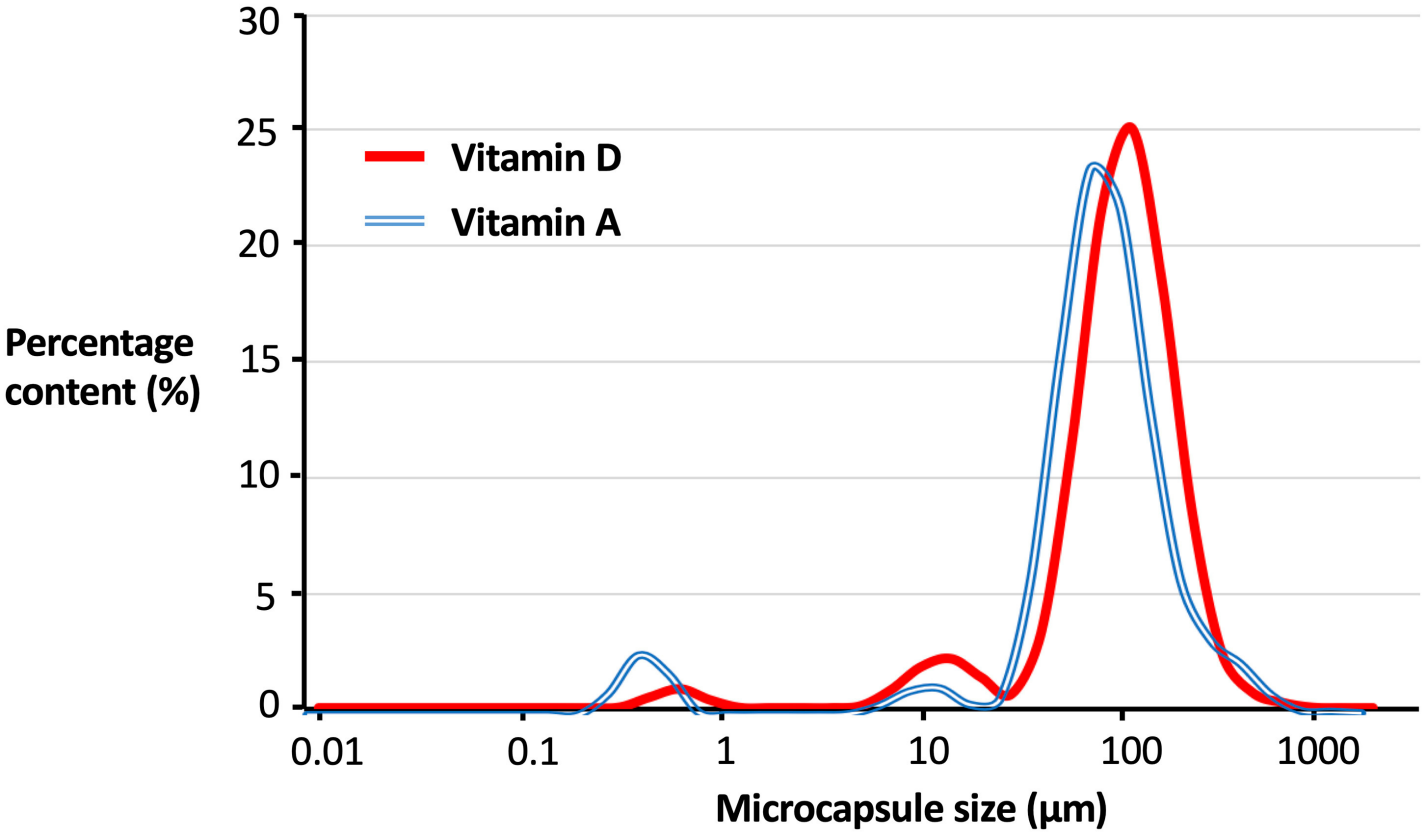
Particle size distribution of vitamin A and D microcapsules. Curves plotted are fitted regressions from a Malvern Mastersizer 3000 (Malvern Panalytical, UK) based off five individual samples. Percentage content (%) is by number of particles. For Vitamin A Residual Standard Deviation (RSD) = 0.4, mean microcapsule size = 120 μm. For Vitamin D RSD = 0.4, mean microcapsule size = 134 μm.

Micronutrient microcapsules containing vitamin A or D were successfully produced and established to have generally homogenous morphology. Scanning electron microscopy (SEM) analyses revealed the microcapsules to be of a consistent spherical shape (Figures 1a,b). Closer examination of the particles showed a roughened surface to the spheres (Figures 1c,d), and imaging following freeze-fracture confirmed the interior of the capsules to be solid without large air pockets (Figures 1e,f). The particles were of neutral buoyancy in saltwater. Laser diffraction particle size analysis indicated that the majority of vitamin A and D microcapsules fell within a size range of 50 to 200 μm diameter. Vitamin A microcapsules had a mean diameter of 120 μm [Residual Standard Deviation (RSD) 0.4 μm] (Figure 2, blue line), and vitamin D slightly larger with a mean diameter of 134 μm (RSD 0.4 μm; Figure 2, red line). For both vitamin A and D microcapsules, there were peaks in particle abundance around 0.5 and 10 μm, but these were very small compared to the main peaks of 50–200 μm microcapsules.

### Nutrient Fortification

**Figure 3:**
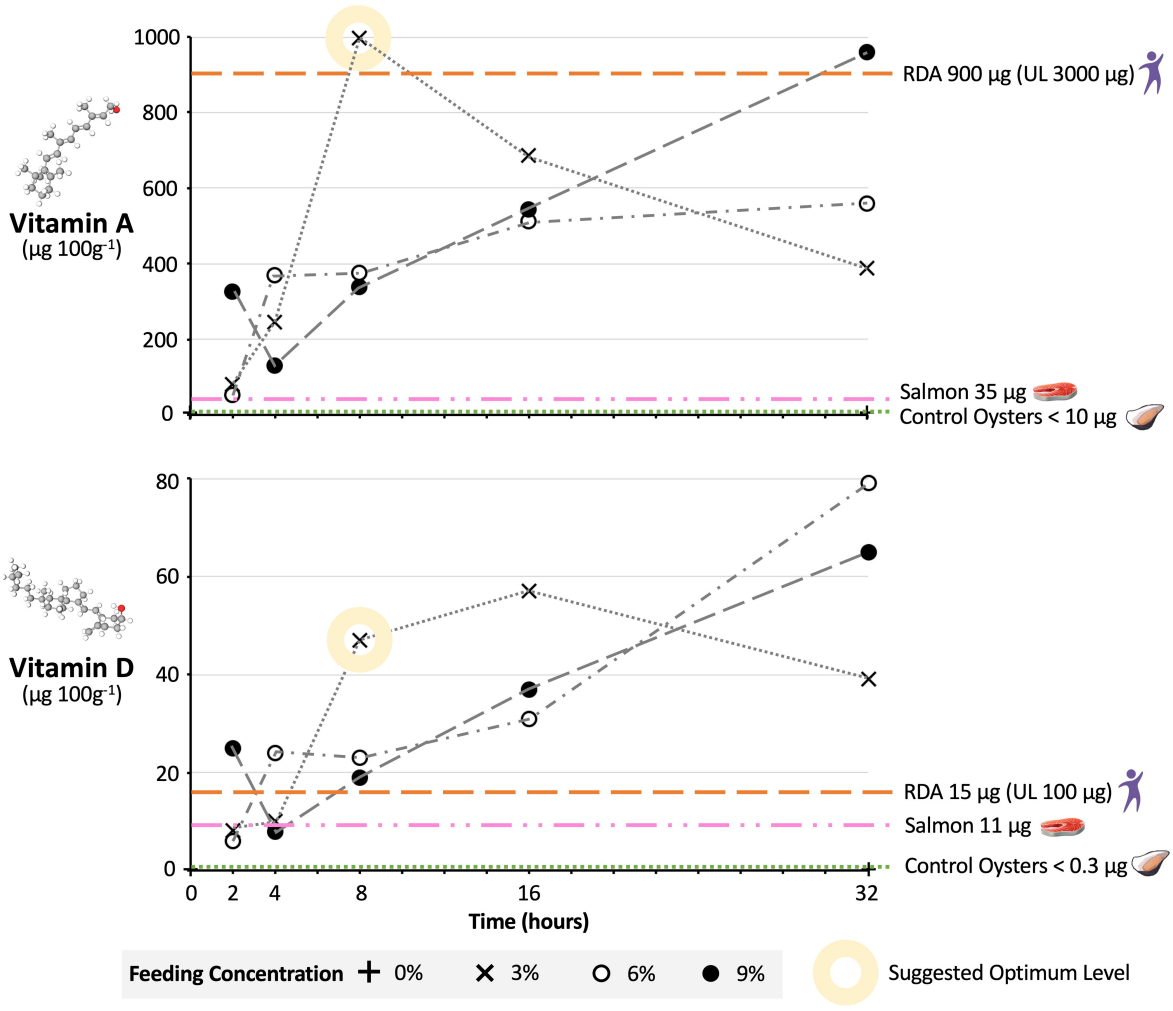
Nutritional uplift of Vitamin A and D in oysters fed fortified microcapsules. Pacific oysters were fed vitamin A and D fortified microcapsules at 3, 6, and 9% dry weight feed per dry weight oyster feeding levels, over time periods of 2, 4, 8, 16, and 32 h. Individual data points are compound analysis values from five oysters individually fed in separate tanks. The relative uncertainty (RU) for vitamin A data points is 12.6% and for vitamin D 19.6%. Vitamin levels in μg are per 100 g of wet oyster. RDA, Recommended Daily Allowance; UL, Upper Daily Limit (41). RDA assumes 100 g portion of oyster meat consumed. Vitamin values for salmon and control oysters are per 100 g wet tissue (16). UK and US regulations respectively stipulate minimum 42- and 44-h depuration periods for bivalves (21).

Pacific oysters successfully consumed microcapsules and this resulted in elevated micronutrient levels in whole-organism tissue samples. In general, increasing the microcapsule concentration and feeding timeframe resulted in higher micronutrient levels in oyster tissue relative to 0% feed concentration controls. This relationship was not completely linear, although the patterns for vitamin A and D microcapsules were the same (Figure 3). The relative uncertainty (RU) for vitamin A data points was 12.6% and for vitamin D 19.6%.

For 3% feed concentrations, oyster vitamin A and D levels after 2 h were 81 and 8.1 μg 100 g^−1^, respectively. At longer timeframes micronutrient levels increased, with the greatest change in micronutrient levels occurring when moving from a 4 to 8-h timeframe. Micronutrient levels peaked at 997 μg 100 g^−1^ for vitamin A after 8 h and at 57 μg 100 g^−1^ for vitamin D after 16 h. At these peaks the percentage of microcapsules in the oysters in relation to the amount added to the tanks (i.e., yield) was 89% for vitamin A and 51% for D. After 32 h levels of both vitamins were lower, at 389 μg 100 g^−1^ for vitamin A and 39 μg 100 g^−1^ for vitamin D.

Oyster micronutrient after 2 h for the 6% feed concentration were similar to the 3% feed concentration, at 52 and 6 μg 100 g^−1^ for vitamins A and D, respectively. However, by 8 h vitamin levels in the oysters on the 6% feed were less than half that of oysters on 3%, at 375 μg 100 g^−1^ for vitamin A and 23 μg 100 g^−1^ for vitamin D. For the 6% feed micronutrient levels did not reach their maximum until the 32-h mark, at 560 and 79 μg 100 g^−1^ for vitamins A and D, respectively. At this point the yield for Vitamin A was 25% and for vitamin D 35%.

The relationship between feeding timeframe and micronutrient levels was broadly similar for oysters on the 9% feed compared to oysters on the 6% feed. Again, micronutrient levels at 8 and 16 h were lower on the 9% feed than on the 3% feed, and on the 9% feed micronutrient levels did not peak until the 32-h mark, with yields of 28 and 19% for vitamin A and D, respectively. The exception was at the 2-h timeframe, where levels of vitamin A at 327 μg 100 g^−1^ and vitamin D at 25 μg 100 g^−1^ were markedly higher than levels on the 3 and 6% feeds.

## Discussion

Microcapsules were developed with appropriate properties to achieve efficient capture and digestion by filter feeding bivalves. The consistent spherical morphology and size range of 50–200 μm, were of a shape and size that *C. gigas* could harvest from the water (31). For both vitamin A and D microcapsules, particles at the peaks around 0.5 and 10 μm likely represent ingredient fragments which can be seen on close inspection of the SEM images (Figures 1a,b). The scarcity of these fragments confirms high purity in the microcapsule samples. The roughened surface structure of the microcapsules will likely have improved their palatability to bivalves (27), and the lack of air pockets helped ensure neutral buoyancy so that the particles remained at the appropriate position in the water column for filter feeders to access (24). These physical properties made the microcapsules an ideal delivery vehicle for the micronutrients in this study and the key component in allowing us to nutritionally fortify bivalves.

Feeding micronutrient microcapsules under depuration conditions led to successful fortification of bivalves, and we suggest that for vitamins A and D an optimum dose regarding feed concentration and timeframe might be 3% for 8 h. After an 8-h timeframe, vitamin A and D levels in oysters were higher on the 3% feed than on the 6 or 9% feed. This relationship is less surprising than first appears; when bivalves are exposed to too much food they will reduce their feeding rate to avoid overloading the filtering system on their gill stacks (31). The only other feed concentrations and timeframe that resulted in comparable vitamin levels to 3% at 8 h were 6 and 9% at 32 h. Feeding at this higher dosage would however not be optimal, representing a wasteful and excessive use of feed resources to achieve a very marginal further increase in oyster vitamin levels. This is demonstrated by the lower yields of the 6 and 9% treatment at 32 h relative to the yield of the 3% treatment at 8 h. We note that the drop-off in micronutrient levels after 32 h for the 3% feed is likely occurring as by this point the oysters have depleted the microcapsules in the tank, and are digesting and excreting the excess vitamin A and D they do not need (42). We therefore suggest that if an 8-h fortification period is used it should be performed at the later stages of depuration to reduce the risk of bivalves excreting nutrients in feces. Optimizing concentration and timeframe are clearly important in ensuring efficient use of resources.

Oysters fortified with vitamins A and D at 3% for 8 h also performed well regarding nutritional value when compared to other foods and the RDAs, providing further support to our suggested optimum dose. In a small portion (100 g, or 3 small or 2 large oysters) of oysters fortified at the 3% 8-h dosage, vitamin A and D levels were 997 and 47 μg 100 g^−1^, respectively. This exceeds the levels in natural oysters (<10 and <0.3 μg 100 g^−1^). More importantly, it far exceeds the levels found in one of the best natural sources of vitamin A and D; salmon (37 and 11 μg 100 g^−1^, Figure 3). Given the highly unsustainable nature of salmon farming relative to bivalve farming and the destructive impact salmon production is having on the environment (43), this offers promise for using bivalves as a planetary health food—good for people and good for the planet (44). In addition, a 100 g serving of oysters fortified at 3% 8-h meets US Department of Health RDAs for vitamin A and D [without exceeding Upper Daily Limits (UL)] (41). Based upon predicted manufacturing, distribution, and implementation costs for the microcapsules, fortification would add just $0.0056 to the cost of a single oyster, which could readily be recuperated through a small additional increase (~0.9%) in oyster retail price. This offers strong hope—for people in deficient populations just two fortified oysters a day could provide them with all their vitamin A and D needs in a highly bioavailable form (10).

### Future Prospects

Looking forwards, there are important steps that can be made by the research and industrial community in order to realize the potential of bivalves and microencapsulation innovations to help tackle micronutrient deficiencies worldwide. Researchers will need to carry out larger laboratory studies with a greater number of replicates to enable quantitative analysis of the individual variation in vitamin uptake by bivalves; such variation is often seen in the fortification of foods including eggs and meat via dietary intervention (45). There is also a need to assess the bioaccumulation of microencapsulated vitamins specifically into bivalve storage tissues, the impact of high-level vitamin accumulation on bivalve physiology, and whether the presence of microcapsules in the bivalve gut promotes the micellarization and absorption of vitamins in the human gut. There is hence a need for proof of concept trials on humans. Future studies would need to feed fortified bivalves to human participants and assess the impact on physical health and blood markers, to establish the true bioavailability of the initially microencapsulated micronutrients to people.

At an international scale, there will be a requirement to tailor the selection of vitamins encapsulated and the microcapsule dosage given, in order to apply the technology to global regions with specific nutritional deficiencies or food consumption patterns. Despite the increased cost of fortified oysters relative to conventional oysters being small (0.9%), and the falling price of oysters with new breeding innovations and the use of fast growing triploids, oysters remain one of the more expensive bivalves (15). It will therefore also be crucial to apply the technology to other bivalve species including mussel and clam species such as *Perna viridis* and *Ruditapes phillippinarum* which are cheaper to farm in many developing regions (15). Completion of these steps will help enable scale-up of micronutrient fortified microcapsules at the commercial level.

There are major economic, sustainability, and health wins that can be made from integrating micronutrient fortified bivalves into our global food system. The ability to use tiny doses of microcapsules to fortify a food organism at its final life stage has major cost advantages. It represents a cheaper option than attempting to fortify other terrestrial animals or fish, which need to be fed fortified feeds for a greater period of their lifespan. Bivalves are also the most sustainable animal food on the planet, with farming having important ecosystem benefits (17), so there are conservation gains that could be made from bivalve aquaculture expanding in place of other meat production. Most importantly, microencapsulated micronutrients combined with bivalve aquaculture can act as a next-level tool to target and tackle nutritional deficiencies worldwide. Just two fortified bivalves a day has the potential to contribute toward saving and improving the lives of over 2 billion people worldwide.

## Conclusions

In summary, this study marks the first successful fortification of bivalves with micronutrients beneficial to human health, using a novel microencapsulated feed supplied at the depuration stage of production. The microcapsules were tailored for optimal size, shape, buoyancy, and palatability to maximize uptake by bivalves. Pacific oysters were selected as a case species, due to their sustainable production and economic importance as the most widely cultivated bivalve globally. Our study found that oysters fed vitamin A or D microcapsules at a dose of 3% over 8 h had increased vitamin content, to the extent that two such oysters would provide enough vitamin A and D to meet human dietary RDAs. Fortification at this level would be highly cost effective and offset by a small (0.9%) increase in retail price.

Further research studies and industry trials are warranted in order to realize the potential benefits of fortified bivalves to the global food system. These can allow us to gain a greater understanding of the inter-individual variation in micronutrient accumulation by bivalves, the bioavailability of delivered nutrients to humans, and the optimum combination of bivalve species, encapsulated nutrients, and fortification dose to help tackle nutrient deficiencies in specific global regions. Taking these steps can provide stakeholders in aquaculture to make an invaluable contribution toward improving the quality and sustainability of our global food system.

## Data Availability Statement

All datasets generated for this study are included in the article/Supplementary Material.

## Ethics Statement

This study used Pacific oysters *Crassostrea gigas*, which are not a regulated organism, and hence no ethical approval was required. The oysters were treated with care during the study.

## Author Contributions

DW led the project and wrote the manuscript. DA contributed to study design, interpretation, and reviewed the manuscript. Both authors gave final approval for publication. All authors contributed to the article and approved the submitted version.

## Conflict of Interest

DA is a Managing Director of BioBullets Ltd. The remaining author declares that the research was conducted in the absence of any commercial or financial relationships that could be construed as a potential conflict of interest.

## References

[1] Ritchie H Roser M Micronutrient deficiency. Our World Data. (2020) 1–21.

[2] WHO Vitamin and Mineral Information System (VMNIS). World Health Organisation. Micronutr Database (2020) Available online at: https://www.who.int/vmnis/database/en/ (Accessed January 20, 2020).

[3] Nair R Maseeh A. Vitamin D: The “sunshine” vitamin. J Pharmacol Pharmacother. (2012) 3:118–26. 10.4103/0976-500X.95506 22629085 PMC3356951

[4] Nimitphong H Holick MF. Prevalence of vitamin D deficiency in Asia vitamin D status and sun exposure in Southeast Asia. Dermatoendocrinol. (2013) 5:34–7. 10.4161/derm.24054 24494040 PMC3897596

[5] G R Gupta A. Vitamin D deficiency in India: prevalence, causalities and interventions. Nutrients. (2014) 6:729–75. 10.3390/nu6020729 24566435 PMC3942730

[6] Man REK Li LJ Cheng CY Wong TY Lamoureux E Sabanayagam C. Prevalence and determinants of suboptimal vitamin D levels in a multiethnic asian population. Nutrients. (2017) 9:1–12. 10.3390/nu9030313 28327512 PMC5372976

[7] Parva NR Tadepalli S Singh P Qian A Joshi R Kandala H. Prevalence of vitamin D deficiency and associated risk factors in the US population (2011-2012). Cureus. (2018) 10:e2741. 10.7759/cureus.2741 30087817 PMC6075634

[8] Myers SS Zanobetti A Kloog I Huybers P Leakey ADB Bloom AJ. Increasing CO_2_ threatens human nutrition. Nature. (2014) 510:139–42. 10.1038/nature13179 24805231 PMC4810679

[9] Barré T Perignon M Gazan R Vieux F Micard V Amiot MJ. Integrating nutrient bioavailability and coproduction links when identifying sustainable diets: How low should we reduce meat consumption? PLoS One. (2018) 13:1–21. 10.1371/journal.pone.0191767 29444098 PMC5812584

[10] Hunter P Nutrition: more than the sum of its parts. The modern craze for dietary supplements is under increasing scrutiny, while biofortified crops look promising in the quest to deliver nutrition in developing countries. EMBO Rep. (2011) 12:307–10. 10.1038/embor.2011.42 21455252 PMC3077257

[11] van Het Hof KH West CE Weststrate JA Hautvast JG. Dietary factors that affect the bioavailability of carotenoids. J Nutr. (2000) 130:503–6. 10.1093/jn/130.3.503 10702576

[12] Hurrell R Egli I. Iron bioavailability and dietary reference values. Am J Clin Nutr. (2010) 91:1461S–7S. 10.3945/ajcn.2010.28674F 20200263

[13] Caballero B Trugo L Finglas P Encyclopedia of Food Sciences and Nutrition. Vol. 1–10. Amsterdam: Elsevier Science BV. (2003).

[14] Horton S. The economics of food fortification. J Nutr. (2006) 136:1068–71. 10.1093/jn/136.4.1068 16549479

[15] FAO FAOSTAT Statistics Database. (2020) Available online at: http://www.fao.org/faostat/en/#data (Accessed January 20, 2020).

[16] U.S. Department of Agriculture. USDA Food Data Central. (2020) Available online at: https://fdc.nal.usda.gov

[17] Willer DF Aldridge DC. Microencapsulated diets to improve bivalve shellfish aquaculture for global food security. Glob Food Sec. (2019) 23:64–73. 10.1016/j.gfs.2019.04.00729291100

[18] FAO Fishery and Aquaculture Statistics. Global aquaculture production 1950-2017 (FishstatJ). Rome: FAO Fisheries and Aquaculture Department (2020) Available online at: www.fao.org/fishery/statistics/software/fishstatj/en (Accessed January 20, 2020).

[19] Gentry RR Froehlich HE Grimm D Kareiva P Parke M Rust M. Mapping the global potential for marine aquaculture. Nat Ecol Evol. (2017) 1:1317–24. 10.1038/s41559-017-0257-9 29046547

[20] Mohamed K Bivalve Mariculture in India - Progress in Research and Development. Central Marine Fisheries Institute, Cochin, Kerala (2015).

[21] FAO Bivalve Depuration: Fundamental and Practical Aspects. Technical Paper, Rome (2008).

[22] Browning LC Cowieson AJ. Vitamin D fortification of eggs for human health. J Sci Food Agric. (2014) 94:1389–96. 10.1002/jsfa.6425 24114770

[23] Seymour W REVIEW: update on vitamin nutrition and fortification in dairy cattle. Prof Anim Sci. (2001) 17:227–37. 10.15232/S1080-7446(15)31634-X

[24] Aldridge DC Elliott P Moggridge GD. Microencapsulated BioBullets for the control of biofouling zebra mussels. Environ Sci Technol. (2006) 40:975–9. 10.1021/es050614+ 16509345

[25] Willer DF Aldridge DC. Microencapsulated diets to improve growth and survivorship in juvenile European flat oysters (*Ostrea edulis*). Aquaculture. (2019) 505:256–62. 10.1016/j.aquaculture.2019.02.072 30890854 PMC6420816

[26] Willer D Aldridge DC. Microencapsulated diets to improve bivalve shellfish aquaculture. R Soc Open Sci. (2017) 4:171142. 10.1098/rsos.171142 29291100 PMC5717674

[27] Luzardo-Alvarez A Otero-Espinar FJ Blanco-Méndez J Microencapsulation of diets and vaccines for cultured fishes, crustaceans and bivalve mollusks. J Drug Deliv Sci Technol. (2010) 20:277–88. 10.1016/S1773-2247(10)50045-5

[28] Knauer J Southgate PC A review of the nutritional requirements of bivalves and development of alternative and artificial diets for bivalve aquaculture. Rev Fish Sci. (1999) 7:241–80. 10.1080/10641269908951362

[29] Langdon C Microparticle types for delivering nutrients to marine fish larvae. Aquaculture. (2003) 227:259–75. 10.1016/S0044-8486(03)00508-8

[30] Grant J Hatcher A Scott DB Pocklington P Schafer CT Winters GV A multidisciplinary approach to evaluating impacts of shellfish aquaculture on benthic communities. Estuaries. (1995) 18:124–44. 10.2307/1352288

[31] Beecham J A Literature Review on Particle Assimilation by Molluscs and Crustaceans. Cefas Contract C2706. Lowestoft: The Centre for Environment, Fisheries and Aquaculture Science (2008). p. 1–18.

[32] Butt MS Arshad MU Alam MS Nadeem MT Bioavailability and storage stability of vitamin A fortificant (retinyl acetate) in fortified cookies. Food Res Int. (2007) 40:1212–9. 10.1016/j.foodres.2007.07.002

[33] Helm M Bourne N The Hatchery Culture of Bivalves: A Practical Manual. Rome: Food and Agriculture Organization of the United Nations (2004).

[34] González-Araya R Lebrun L Quéré C Robert R The selection of an ideal diet for *Ostrea edulis* (L.) broodstock conditioning (part B). Aquaculture. (2012) 362–3:55–66. 10.1016/j.aquaculture.2012.06.029

[35] Thompson JN Hatina G Maxwell WB. High performance liquid chromatographic determination of vitamin A in margarine, milk, partially skimmed milk, and skimmed milk. J Assoc Off Anal Chem. (1980) 63:894–8. 7400091

[36] Dias MG Camões MFGFC Oliveira L Carotenoids in traditional Portuguese fruits and vegetables. Food Chem. (2009) 113:808–15. 10.1016/j.foodchem.2008.08.002

[37] Thompson JN Hatina G Maxwell WB Duval S. High performance liquid chromatographic determination of vitamin D in fortified milks, margarine, and infant formulas. J Assoc Off Anal Chem. (1982) 65:624–31. 7096244

[38] Bogard JR Thilsted SH Marks GC Wahab MA Hossain MAR Jakobsen J Nutrient composition of important fish species in Bangladesh and potential contribution to recommended nutrient intakes. J Food Compos Anal. (2015) 42:120–33. 10.1016/j.jfca.2015.03.002

[39] Peng X Wood CL Blalock EM Chen KC Landfield PW Stromberg AJ. Statistical implications of pooling RNA samples for microarray experiments. BMC Bioinformatics. (2003) 4:26. 10.1186/1471-2105-4-26 12823867 PMC166151

[40] Heffernan AL Aylward LL Toms L-ML Sly PD Macleod M Mueller JF. Pooled biological specimens for human biomonitoring of environmental chemicals: opportunities and limitations. J Expo Sci Environ Epidemiol. (2014) 24:225–32. 10.1038/jes.2013.76 24192659

[41] US Department of Health & Human Sciences Fact Sheet for Health Professionals. National Institutes of Health, Office of Dietary Supplements (2020) Available online at: https://ods.od.nih.gov

[42] Gosling E Marine Bivalve Molluscs, Second Edition. Ireland: Wiley Blackwell (2015).

[43] Bostock J McAndrew B Richards R Jauncey K Telfer T Lorenzen K. Aquaculture: global status and trends. Philos Trans. (2010) 365:2897–912. 10.1098/rstb.2010.0170 20713392 PMC2935128

[44] Willett W Rockström J Loken B Springmann M Lang T Vermeulen S. Food in the anthropocene: the EAT-*Lancet* Commission on healthy diets from sustainable food systems. Lancet. (2019) 393:447–92. 10.1016/S0140-6736(18)31788-4 30660336

[45] Leeson S Caston LJ Vitamin enrichment of eggs. J Appl Poult Res. (2003) 12:24–6. 10.1093/japr/12.1.24

